# Utility of dairy microbiome as a tool for authentication and traceability

**DOI:** 10.1515/biol-2022-0983

**Published:** 2024-10-30

**Authors:** Maria V. Alvanou, Dimitrios Loukovitis, Katerina Melfou, Ioannis A. Giantsis

**Affiliations:** Division of Animal Science, Faculty of Agricultural Sciences, University of Western Macedonia, 53100, Florina, Greece; Department of Fisheries and Aquaculture, School of Agricultural Sciences, University of Patras, 30200, Messolonghi, Greece; Department of Animal Science, Faculty of Agriculture, Forestry and Natural Environment, Aristotle University of Thessaloniki, 54621, Thessaloniki, Greece

**Keywords:** sheep, goat, microbiome, authentication, PDO, PGI, fraud

## Abstract

Milk microbiome contributes substantially to the formation of specific organoleptic and physicochemical characteristics of dairy products. The assessment of the composition and abundance of milk microbiota is a challenging task strongly influenced by many environmental factors. Specific dairy products may be designated by the Protected Designation of Origin (PDO) and Protected Geographical Indication (PGI) labeling, which however, occasionally fail to differentiate them according to specific quality characteristics, which are defined by different microbiota-driven reactions. Combining the above limitations, the scope of the present study, was to summarize the existing information toward three main issues. First, to assess the influence level of the diet type and grazing to rumen–GI tract, mammary gland, and udder microbiome formation in ruminants. Second, to discuss the factors affecting milk microbiota, as well as the effect of the endo-mammary route on milk microbial taxa. Lastly, to evaluate “milk microbiome” as a tool for product differentiation, according to origin, which will contribute to a more robust PDO and PGI labeling. Although the limitations are still a matter of fact (especially considering the sample collection, process, evaluation, and avoidance of its contamination), significant progress has been made, regarding the identification of the factors affecting dairy products’ microbiota and its core composition. In conclusion, although so far not totally efficient in dairy products molecular identification, with the progress in soil, water, plant, and animal host’s microbiota assembly’s characterization, microbiomics could provide a powerful tool for authentication and traceability of dairy products.

## 1 Introduction

Ruminants represent an important part of global agrifood sector, contributing greatly to agroecology [1]. They are unique in their ability to convert inedible feed (plant cell wall carbohydrates) into high-valued protein such as meat and dairy products. Food by-products [2], forages [3], and non-protein nitrogen [4] can be efficiently included in their diet. It is estimated that the increase in livestock products demand will double by 2050, in line with the global population increase [5,6]. The livestock sector provides employment for more than one billion people while at the same time contributes to 40% of agricultural gross domestic product [7].

Ruminants, as mammalian herbivores are unable to produce cellulolytic or hemi-cellulolytic enzymes for degradation of ingested plant material. Their adaptation toward the degradation is based on the development of a symbiotic relationship with microbial communities (such as bacterial, fungi, and protozoa) for performing digestion [8]. Generally, diet and nutrition of ruminants has been proposed as the main driving force toward microbial community composition in rumen [9], affecting nutritional value of products [10]. Nevertheless, apart from the positive effects of rumen fermentation toward food production, it has also a negative impact toward environment as it is associated with emissions of greenhouse gases [11,12] and nitrogen [13]. As a result, many scientific papers focus on the understanding of rumen microbial composition, both for production improvement and reduction of environmental impact [14].

Recent evidence proposes that microorganisms inhabiting gut, namely “gut microbiome,” exhibit a main role toward host physiology [15]. Rumen forms an anaerobic environment, where a complex microbial community, consisting of bacteria, archaea, protozoa, and fungi, grow, with bacteria being the most abundant [9]. This complex rumen microbiota structure has been described as “the most elegant and highly evolved cellulose-digesting system in nature” [16]. For instance, 7,416 ruminal microbial taxa have been identified in lactating Holstein cows, with rumen microbiome being dominated by Bacteroidetes, Firmicutes, and Proteobacteria phyla, while the most abundant genera were *Prevotella*, *Succiniclasticum*, and *Prevotellaceae UCG-001* [17]. Furthermore, a strong correlation has been demonstrated between abundance of various bacterial members of the rumen microbiome and physiological parameters of the host, such as milk yield and composition [18]. The organism welfare significantly influences the production rates as it modulates microbiological composition of the rumen [19].

Rumen–gut microbiome seems to be affected by many factors including age, diet, welfare, physiological condition, and surrounding environment. In addition, common mistakes, such as inadequate living conditions and improper diet, often take place during the intensified breeding of ruminants with possible negative impacts toward digestive tract microbiota leading to health status deprivation [20,21]. Apart from their substantial contribution toward health status, the proper composition and quantity of microbiota ensures homeostasis of organism, while in parallel influences the level of methane production [22,23]. Despite the fact that the two-way communication between the microflora of the gastrointestinal tract and the central nervous system is known [24], questions still remain regarding the existence of microbiota transfer through an entero-mammary route [25,26].

Apart from its multiple roles for the organism, microbiome is suggested to be responsible for the final quality characteristics of many agrifood products such as dairy and wine, on account of main microbiota-driven reactions. Recently, the microbiome has been used as a tool/marker for studying the geographic origin of various food products, including shellfish, such as mussels, oysters, and clams [27], *Vitis vinifera* through the investigation of soil and root-associated microbiome [28], while it has also revealed associations among different terroirs and wine varieties in Cyprus, from the stages of pre- until post-fermentation [29].

Contrariwise, for the dairy sector, microbiome investigation represents a more complex task. For instance, in cheese and especially for rennet-coagulated ones, the rennet, starter, and adjunct microbial cultures are added to accelerate ripening process. The majority of cheeses consist of four main ingredients: milk, rennet, microorganisms (usually starter cultures of lactic acid bacteria [LAB)]), and salt [30]. In dairy LAB two main biotechnological groups are included: starter LAB (SLAB) and non-starter LAB (NSLAB) [30]. The SLAB usually originates from natural starter cultures (NSC) and commercial starter cultures. These bacteria cultures are added in the beginning of the cheese making process and contribute to the pH reduction which helps in milk coagulation before proceeding to the next step where the addition of rennet or another coagulating agent takes place. Apart from that, SLAB also participates in the development of desirable flavor compounds in cheese [31]. NSCs are retained from back slopping, a technique where the whey from the successfully manufactured cheese batch can be retained for future use as the inoculum or starter culture for the next batch [32,33]. One of the main goals of NSC is to keep the genetic variability and the biodiversity of the microorganisms existing in cheese [34]. These actions affect nutritional, technological, and sensory properties of cheese, whereas they modify the final cheese microbiota profile as well [35]. Regarding milk, there are many factors that may affect its microbial composition, including host and environment, as well as the type of strategies followed for sampling, sequencing, and statistical analysis (i.e., various databases available), which may add bias and lead to distortion in milk microbiota analysis [25]. Therefore, from the limited so far existing studies, one interesting point inferred, is the effect of NSC on the final cheese’s microbiota, which in turns influence the flavor profile and can provide some information regarding its origin.

The development of powerful sequencing tools allows the successful investigation of the key factors affecting ruminant’s microflora and the role of microbiome toward health and production of the animal [19]. More specifically, the methods that are culture independent (i.e., 16S RNA sequencing ribosomal RNA, shotgun DNA-seq or RNA-seq which targets entire nucleotide sequence, and metatranscriptomics which enables the detection of changes in gene expression) are gaining more and more attention [19]. Additionally, microbiome has gained more attention recently as a tool for deep analysis, potential characterization, and authentication of several products [36]. Nevertheless, there is still no coherence in information regarding the influence of the ruminal, gut and mammary gland, and udder microbiota in the milk microbiota. Keeping this in mind, the present study attempts to summarize the current information toward three main components:

(a) to evaluate how the diet-type and grazing affect ruminant’s ruminal-intestinal microbiome,
(b) to explore if and how the ruminal-intestinal, mammary gland, and udder microflora affect milk, and lastly
(c) to examine if the produced milk can provide information regarding its origin by investigating its microbiota.

Hence, the main scope of the study is to connect the aforementioned components into a simpler question: *Can the milk and/or cheese microbiome synthesis provide information regarding the origin of the dairy products and at what extent the animal’s diet type and composition affects it?*

## 2 Influence of animal diet, grazing, and “freedom of choice” on rumen–GI tract and udder microbiota

The crucial role of diet in rumen and gut microbiota formation and activity is well documented [10,37] and may shift microbial community composition [38–42]. Alterations in the composition of rumen microbiota which regulate animal’s metabolism, impact host’s phenotype as well [43]. Feeding regimes and feed additives may influence the composition and functions of rumen’s microbiome. As a result, the impact of diet on the microbiome and hence to the product quality characteristics is of high interest. As reported by Wang et al. [44] artificial pasture grazing (an artificial grassland with *Medicago sativa*, *Poa pratensis*, and *Bromus riparius* was prepared) influenced muscle metabolites by modulating rumen microflora. Furthermore, high-energy diets are found to alter rumen microbiota composition, by increasing the abundance of *Quinella*, *Ruminococcus* 2, *Eubacterium*, *Coprostanoligenes*, and *Succinivibrionaceae* UCG-001, which are associated with carbohydrate metabolism in rumen [43], whereas indoor feeding regimes alter the abundance of rumen bacteria (*Christensenellaceae* R-7 group, [Eubacterium] *Coprostanoligenes* group, *Methanobrevibacter*, *Ruminococcus* 2, and *Quinella*) which influence muscle metabolism [45]. It can thus be concluded that host phenotype and metabolism can be altered according to different feeding regimes and dietary supplements. Based on the aforementioned studies, it is suggested that diet can affect rumen microbiota and product quality.

As the influence of diet on ruminal and intestinal microbiota is widely known, the dietary diversity in ruminants is receiving more and more attention [46–49]. In the context of dietary diversity, the “freedom of choice” regarding their diet and how this component can affect animal production is also included. It was observed that by choosing and mixing their diets, animals can reciprocate toward their needs, while at the same time they can self-medicate by consuming and digest plants with specific secondary compounds (PSC) that are associated with nutraceuticals, pharmaceuticals, and prophylactic benefits [50]. Dietary diversity in contrast to dietary monotony can improve ruminant’s welfare by enhancing hedonism and eudaimonism, while at the same time reduces stress levels [51]. Furthermore, diversity in dietary sources can influence the microbial-rumen–gut–brain axis, which is related with alterations in mood, emotions, cognition, and behavior both in humans [52] and farm animals [53]. Hence, dietary monotony can impact dietary preference and eating behavior in ruminants, whereas, on the other hand, grazing can lead to higher dietary diversity by providing animals the freedom of choice. In addition, grazing has a positive effect toward sustainable livestock production and animal welfare [54]. Although there are several studies on diet influence on ruminants’ microbiota [55] and microbial alterations over the transition from forage to concentrate diets [38,56], gaps still exist regarding the effect of the dietary diversity, achieved by grazing, on the microbiome. Belanche et al. [57] revealed that grazing may lead to an increase in the concentration, diversity, network, and abundance of key microbes involved in cellulolysis, lactate production, and methylotrophic archaea populations. Additionally, a core microbiota composition was observed which consisted of 34, 9, and 13 genera for bacteria, methanogens, and fungi, respectively, which were common among sheep independent of grazing or non-grazing [57].

Considering the strong effect of diet on rumen microbiome, it is not surprising that there are plenty of studies investigating the addition of many additives in order to control the gut–rumen axis complex composition. Under this prism, one of the main targeting actions that can cause manipulation of rumen microbiome is the improvement of the animal’s overall health. Animal health is in line with the better nutritional composition of animal products, regarding the lipid and fatty acid content/composition, as well as with the prevention of pathogen transfer in the food supply chain [15]. Regarding additives, plant-derived essential oils (EOs) obtained from the secondary metabolism of plants associated with the odor and spices of plants, often exhibit antimicrobial activity [58]. Depending on the dose and type of administration, EOs may alter gut microbiome, improve rumen fermentation efficiency, and support small ruminant-derived products. The impact on the abundance and composition of rumen’s microorganisms can in turn alter rumen function and green house gases production, by increasing ruminal absorption of total volatile fatty acid (VFA) concentration, better feed conversion ratios and improvement of gut immune responses, integrity of intestinal barrier, and growth performance rates [59].

Udder homeostasis is influenced by many management practices including housing conditions, nutritional status, and antimicrobial usage [60]. Among the nutritional factors, it was observed that feeding conditions, mainly indoor and grazing, pose a pivotal role toward the formation of the microbiome in mammary glands of dairy cows. More specifically, microbial diversity of udder skin tissues was higher during grazing compared to feeding period [61]. Furthermore, dietary supplementation with bamboo leaf extract was found to affect udder and milk microbiome in dairy cows, highlighting the important role of diet in udder microbiome [62].

Based on all the above information, it can be concluded that dietary interventions influence rumen and gut microbiota and may have an impact on dairy products. However, the influence of pasture grazing on milk microbiota and, subsequently, to the microbiota associated with the milk processed and final products, nutritional value, and consumer health represents a very challenging task [61]. These difficulties mostly concern environmental conditions, management practices, animal genetics, seasonality, hygiene, and feedstuffs [63].

## 3 Impact of rumen, gut, mammary gland, and udder microbiota on milk production and quality

Contrarily to the negative impacts of methane production, rumen and gut microbiomes contribute toward the break down and digestion of the feed consumed by the animal, controlling the production efficiency [64]. More specifically, the complex rumen microbiome facilitates the digestion of complex feed substrates that are high in fiber concentration, into VFA and microbial proteins. These microbial proteins are essential for animal growth, maintenance, and lactation processes [65]. Within the context of lactation, microbiota composition of rumen affects the variation of milk fatty acids in dairy cows [66]. Moreover, high-yielding Holstein cows possess significantly enriched ruminal microbiome, being associated with effective cellulose degradation and increased milk production, compared to low-yielding animals [67].

Apart from milk quality characteristics, the influence of the rumen–gut axis microbiome on milk microbiome still remains unexplored. Although occasional “core” microbiome patterns have been observed in rumen [10], efforts have been also carried out to characterize the potential core microbiota patterns in the mammary glands [25]. Despite the significant progress conducted, the assessment of the composition and abundance of microbial communities in milk still remains a challenging task, as there are many difficulties in the collection of a non-contaminated representative milk sample. Regarding human milk for instance, obstacles still exist in the collection of a sample with low bacterial biomass, non-contaminated with reagent DNA [68], while a standardized method is missing [69], leading to a wide variation in the results of microbiota estimation [68]. The main identified problems in human milk, which has been studied more extensively, may also be applicable to the study of the microbiome of ruminant milk. Indeed, based on previously published studies, similar issues have been reported in ruminant’s milk microbiota analysis [70–72].

Previous studies additionally indicated the significant influence of farming system on special bacterial traits observed in bovine milk, between indoor housing and pasture [61,73,74]. More specifically, grazing is proposed to increase counts of all bacterial categories [73] and the relative abundances of lactic acid and other probiotic bacteria (Propionibacteria and Bifidobacteria), while decreasing at the same time the abundances of spoilage bacteria [75]. Moreover, differences in farming systems and dairy facilities seem to influence the process of cheesemaking and final product quality, which can serve as an index of the origin of dairy products authentication [76]. However, considering that the investigation of milk microbiome represents a very complex task, apart from the farming system, it may be affected by the season and the lactation stage [75], environmental exposure [77], and contamination [78] making the authentication process even more difficult.

Among the environmental factors that seem to affect microbiota composition in milk is the geography. Kumar et al. [79] observed distinct microbiota patterns in human milk in women from different countries. Regarding bovine milk, factors associated with management practices and environment of the farm are proposed to affect milk microbiota patterns [80,81], whereas in other studies a core microbiome in bovine milk failed to be confirmed [82].

There are some hypotheses regarding the existence of an endogenous entero-mammary pathway by which some bacterial species may be transferred from gut to mammary gland and finally to the produced bovine milk [82,83]. This transfer can be possible considering the ability of some microbiomes to leave the intestinal environment, move through the lymph nodes and reach the mammary gland as a final destination. This endogenous route is supported by a plethora of studies regarding human [84–88], sows [89], cows [83], and mice, while more recent studies confirm this specific route with well-established evidence as reviewed by Selvamani et al. [90]. Nevertheless, the aforementioned route is still considered a theory with other studies expressing opposite opinions [26,60], claiming that the presence of gut-associated bacteria in milk microbiome does not necessarily confirm the endo-mammary route hypothesis, since these microorganisms are also present in the dairy environment [60].

**Figure 1:**
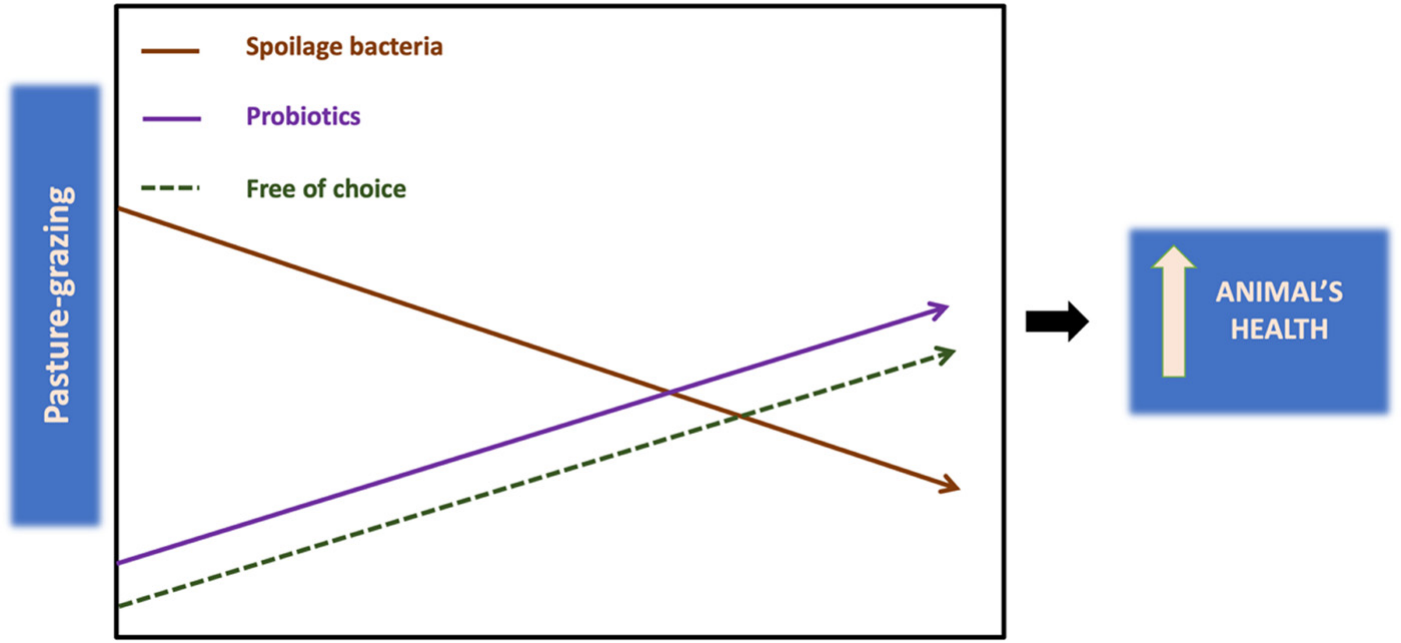
The rumen–gut axis microbiome effect on animal health status. Pasture-grazing practices provide the animals with the “free of choice” option, while it may lead to microbiota “dysbiosis” reduction, by eliminating spoilage bacteria in the gut. Together with the inclusion of other feed additives such as probiotics the above components can contribute to improve of animal’s health.

Rumen–gut axis microbiome is strongly associated with productive performance and health status of animals (Figure 1). Based on evidence mentioned previously, there is a connection of gut and mammary gland microbiota through an endo-mammary route, which seems to have an influence on milk microbiome as well. Recently, dietary probiotics and other feed additives are gaining more and more attention toward the development of a rumen–gut favorable for the animals. By providing the animals with high-quality feedstuffs and by finding alternative solutions to avoid antibiotic administration, disruption of the normal microbiota in ruminant’s rumen and gut, and microbiota “dysbiosis” can be avoided [91].

## 4 From raw milk microbiome to cheese microbiome and its characteristics

Protected Designation of Origin (PDO) and Protected Geographical Indication (PGI) are quality labels established by the European Union for traditional products’ protection, which secure their economic benefits and origin by preventing commercial fraud (European Commission, 2013) [92]. Plenty of methodologies have been developed for dairy products characterization such as infrared spectroscopy [93], nuclear magnetic resonance spectroscopy [94], high-performance liquid chromatography [95], gas chromatography [96], solid phase microextraction, purge and trap [97], and more recently high throughput sequencing technologies [36].

Raw milk, by definition, is “the milk produced by animals (such as cows, sheep, goats, and buffaloes) which has not been heated above 40°C and subjected to any other treatment processes that have an equivalent effect on the milk-associated microbial community” [98]. There is strict E.U. regulation regarding PDO products for raw milk cheesemaking, as the unique microbial communities that exist in raw milk, provide special organoleptic characteristics in the fermented product in close association with the geographical area [99].

Microbiome has been used as a tool to distinguish geographical regions by giving different properties to various agricultural products, such as the final wine product, suggesting it as a potential biomarker [28,29]. Similarly, microbiome has been proposed as a marker for geographical origin identification of fresh seafood [27]. Similarly, it is of high interest to investigate the potential role of microbiome toward the traceability of dairy products.

In raw milk, there are many factors affecting resident microbial community (Section 3) (e.g., diet) [100]. For instance, dietary supplementation with omega-3 fatty acid has been proposed to reduce the pathogenic microbiota in the bovine milk [101]. Furthermore, the use of back-slopping is also beneficial for the raw milk cheeses. Back-slopping refers to the use of natural whey cultures (NWCs) that consist of fermented milk with a complex microbial community from the raw milk. Owing to their nature, NWCs are characterized of high variability according to different production sites and specific environmental factors [102]. Quality characteristics of dairy products, such as aroma, texture, and flavor are closely related to microbiome diversity and abundance, comprising the result of the substances produced from the chemical reactions of carbohydrates, proteins, and lipids [103–105].

Surprisingly, based on a comprehensive analysis of Italian raw milk cheese microbiota, organoleptic qualities of cheeses with the same PDO label differed between manufacturers. Resident microbial populations constitute a more robust factor that controls the final product’s quality characteristics. In line with this, it has been suggested that the PDO label should be revisited for a deeper understanding of the factors leading to final organoleptic characteristics of dairy products [99]. However, the above study received strict criticism regarding the experimental approach, the interpretation of results, and for citation of biased literature [106]. A recent study revealed that production origin can be correlated with the presence of specific metabolites in buffalo’s milk from PDO and non-PDO regions in Italy, with some specific metabolites detected in both the metabolome of unprocessed milk and corresponding mozzarella cheese in buffalo (i.e., talopyranose and *N*-acetyl glucosamine) [107]. These metabolites may also be the result of microbiome-driven chemical reactions, which however might need further investigation. An assay of the core microbiome and bacterial diversity in buffalo milk suggested a high farm management effect on the microbiome [108]. Farming conditions were also found to be the main driving force for the overall microbiome richness in cow’s milk [109]. In addition, when PDO feta cheeses from two geographic regions of Greece were analyzed, two distinctive microbiota fingerprints were observed. The starter culture bacteria species had, however, a strong influence on the final microbial composition [110]. Overall, the main factors responsible for cheese microbiota formation are secondary structuring factors, such as dairy product type, geographical area, and ripening practices, highlighting the contribution of biogeography and PDO-specific know-how of the final product [111].

By combining the strong influence of NWCs on cheesemaking and organoleptic characteristics of the final product with the unique characteristics of each NWCs, which is in strong association with a complex microbial community of raw milk from each region, it would be of great interest to evaluate if this could operate as a tool for origin identification. Toward this direction, starter cultures are pivotal for the final dairy product and the use of the selected starter/non-starter LAB cultures are occasionally proved to successfully stabilize PDO cheese production [112]. A very recent study proposed that PDO artisanal blue-veined cheeses are characterized by dominant components of the cheese microbiome that can provide very useful information for its authentication [113]. A summarization of the above applications is presented in Table 1.

**Table 1:** Scopes of the present study, supported by the corresponding findings

Applications	Findings	References
**Application – factor 1**		
Diet-type and grazing influence on ruminant’s ruminal-intestinal microbiome	Feeding regimes and feed additives may influence the composition and functions of rumen’s microbiome Artificial pasture grazing influenced muscle metabolites by modulating rumen microflora High-energy diets are found to alter rumen microbiota composition associated with carbohydrate metabolism in rumen Indoor feeding regimes can alter the abundance of rumen bacteria and influence muscle metabolism Dietary diversity in contrast to dietary monotony can improve ruminant’s welfare by enhancing hedonism and eudaimonism, while at the same time reduces stress levels EOs may alter gut microbiome, improve rumen fermentation efficiency, and support small ruminant-derived products	[43]
		[44]
		[43]
		[45]
		[51]
		[59]
**Application – factor 2**		
Influence of the ruminal-intestinal, mammary gland and udder microflora on milk microbiota	There are some hypotheses regarding the existence of an endogenous entero-mammary pathway by which some bacterial species may be transferred from gut to mammary gland and finally to the produced bovine milk Some microbiomes leave the intestinal environment, move through the lymph nodes and reach mammary gland as a final destination Opposing opinions claim that the presence of gut-associated bacteria in milk microbiome does not necessarily confirm the endo-mammary route hypothesis, as these microorganisms are also present in the dairy environment	[82]
		[83]
		[60]
**Application – factor 3**		
Milk and dairy microbiota as a tool for authentication and traceability	NWCs are characterized of high variability according to different production sites and specific environmental factors Farming conditions were also found to be the main driving force for the overall microbiome richness in cow’s milk The starter culture bacteria species had, however, a strong influence on the final microbial composition	[102]
		[109]
		[110]

## 5 Limitations and future perspectives

Loss in abundance and diversity of microbiome species is a common finding under several stressful conditions including diseases. Dietary substances provide different substrates for each microbial species enhancing its growth. A greater composition diversity diet may lead to a more diverse microbiome [114]. Furthermore, the diet type, apart from rumen–gut microbiome, also affects udder and mammary gland microbiota in ruminants. However, farming conditions seem to drastically affect raw milk composition [109]. In particular, individual farms [115,116] and different dairy plants lead to different production and quality efficiency of cheesemaking [117].

High-throughput sequencing technologies enhance the ability to track and understand the complex ecosystem of microorganisms in dairy products and thus improve their stability and quality through precision fermentation [118]. However, so far, there is a gap in identification of a core milk microbiome due to many existing difficulties. More specifically, there is a lack of standardized methods for collection, process, and evaluation of milk samples [26].

Pasteurization can influence autochthonous raw milk microbiota while inactivate microbial-produced antimicrobial compounds, by modestly affecting richness and significantly affecting composition [119]. The use of NWCs will provide characteristics that are unique for each region and are expected to further support the traceability of the final product by producing natural adjunct cultures [120], which is already found to be the most important factor for microbiome composition in the case of mozzarella PDO cheese [121].

Along with microclimate, native microbiota fauna in specific geographic regions, included in animals’ diet by grazing, constitute factors that also contribute to the unique characteristics of the final product with PDO and PGI labeling. However, regarding dairy products, there are also other significant components affecting the special organoleptic properties (Section 4). Thus, microbiome investigation, as a potential tool for identifying the product origin and to keep the quality and stability of the final product, will be beneficial for both consumers and producers. A deep understanding of all the aforementioned factors is expected to shed light on the milk microbiome composition and, in a more general sense, on cheese microbiota that give dairy products their uniqueness. More specifically, the investigation of the native fauna effect on mammary gland microbiome through the endo-mammary route, as well as to udder microbiome, which is in contact with native fauna via soil of each region is of great interest. This is especially important in regions characterized by high biodiversity, contributing to rumen–GI tract microbiome, often influenced by the farming conditions as well. In this context, the “free of choice” diet accomplished by grazing can provide access to animals in plants with secondary metabolites such as EO. For this reason, there are two possible advantages toward microbiome with the “free of choice” diet: (a) improvement of overall well-being and (b) self-medicate by consuming and digest plants with PSC that are associated with nutraceuticals, pharmaceuticals, and prophylactic properties. Thus, the potential elimination of the effect from other environmental factors such as the farming conditions, which also have an effect on the typicity of the final product, might lead to a more stable core microbiome of the dairy products for a specific geographical region. At the same time, common NWCs in each specific region that highly affect the fermentation for cheesemaking provide both higher quality and stability.

## 6 Conclusions

**Figure 2:**
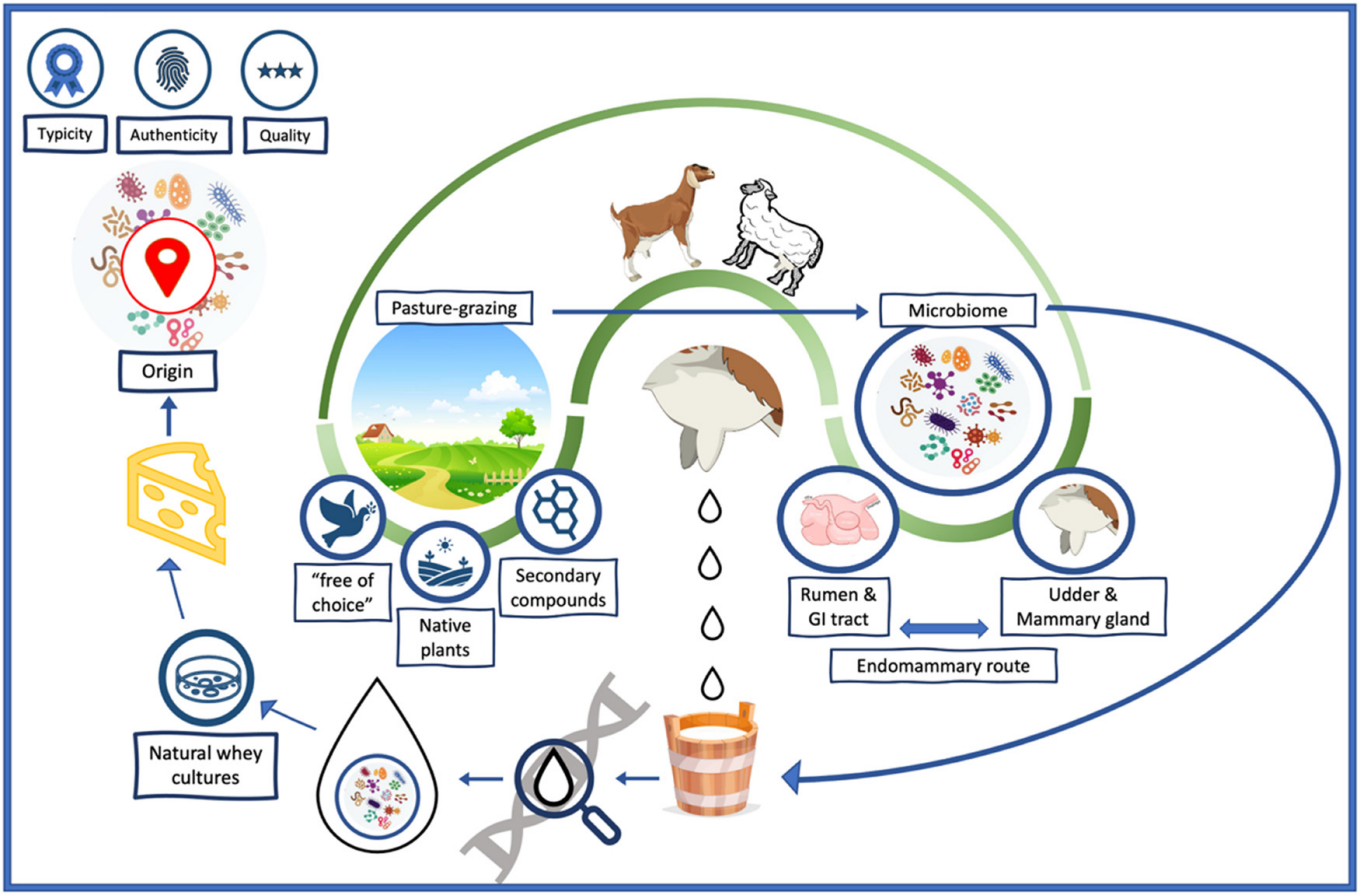
Proposed model factors affecting microbiome in small ruminants and its potential use in authentication. Implementation of pasture-grazing techniques provides the animals a “free of choice” option, and access to unique flora of each region. Apart from the specific unique characteristic of plants from each region, animals are also consuming the secondary metabolites of these plants. The above factors have been shown to influence the rumen and GI tract microbiome, as diet is one of the main driving factors shaping the formation and composition of the microbiome in these organs. Further, the rumen and GI tract microbiome can possibly influence udder and mammary gland microbiome through the endo-mammary route. The milk microbiome included in the milk from these animals will be influenced from all the previous components. Finally, with the addition of NWC – which has been found to be a major factor contributing to final cheese organoleptic characteristic – the dairy products will be characterized by specific microbiota diversity and composition operating as an additional tool for the PDO and PGI products identification.

Although PDO and PGI products are characterized by high quality, they are non-standardized, while their organoleptic characteristics are influenced from many factors during milk production and processing. According to the proposed model of Figure 2, microbial populations existing in raw milk constitute one of the main driving factors for the chemical reactions leading to the formation of specific compounds responsible for the unique organoleptic characteristic of the final product. Among the factors contributing to the formation of milk microbiota the farming conditions, the udder microbiome of the animal, the rumen–GI tract microbiome, the animal’s physiological state, and diet are included. Regarding the rumen–GI tract microbiome contribution to the milk microbiota abundance and composition, it is mainly accomplished by an endo-mammary route, though the extent of the above contribution is still under investigation. Foraging and “free of choice” diet types, through increasing the overall wellbeing and eudemonic of the ruminants, have a substantial contribution to the complexity of animal’s microbiota regarding diversity and abundance microbiome increase (Figure 2). Furthermore, the type of grazing system affects the udder and teat microbiota which in turn, affect the microbiome in raw milk.

Thus, in conclusion, there are three key steps toward the precise geographic characterization of a dairy product. First, the diet type which leads to the formation of the unique microorganism populations in rumen–GI tract and in the udder of the ruminants. Second, the effect of this unique microbiota complex both from rumen–GI tract and from udder on the milk microbiome (considering the existence of the entero-mammary route). Lastly, the contribution of the above two components toward characterization of a raw milk that has been produced from animals having the same diet type from the same geographical region. The combination of the above three steps will lead to final products with specific characteristics that can be traced by investigating the microbiota pattern in the final product, giving information for the origin and avoiding food frauds. Generally, authentication of the origin of the dairy products is crucial, as sometimes the food labeling can be substituted or misrepresented, for achieving an economic gain. The above methodologies may contribute to the detection of possible food frauds by developing anti-fraud services [36,122]. Under this prism, particularly for dairy products under the PDO and the PGI nomination, molecular microbiome is suggested to be further investigated as a tool, in order to better characterize the products according to their organoleptic characteristics and origin.
